# Retraction: Replication, Neurotropism, and Pathogenicity of Avian Paramyxovirus Serotypes 1–9 in Chickens and Ducks

**DOI:** 10.1371/journal.pone.0244178

**Published:** 2020-12-14

**Authors:** The PLOS ONE Editors

After this article [1] was published, concerns were raised about results reported in Figures 3 and 7.

Specifically:

- The APMV-1 (BC) and APMV-8 panels of Figure 3A in [1] appear similar to the APMV-1 (BC) and rAPMV-4/ Fc SV panels, respectively, of Figure 5 in [2]. The authors confirmed the same APMV-1 (BC) image was used in both figures; they stated that the two experiments were conducted simultaneously and that the same APMV-1 control applied to both. Regarding the APMV-8 [1] and rAPMV-4/ Fc SV [2] panels, the authors commented that data reuse may have resulted from an error in figure assembly.
- The Mock-infected panels of Figure 7A and 7B in [1] appear similar to the Uninfected Trachea panel in Figure 5A and Uninfected Lung panel in Figure 5B, respectively, in [3]. The authors confirmed that the same controls were used in the indicated figures. They noted that the experiments were conducted at nearly the same time and that controls were reused because the number of animals was limited.

The authors noted that the original data underlying the results reported in this article are no longer available due to the amount of time that has passed since the experiments were conducted. There are six instances where the Results section refers to data not shown, and the data underlying those statements are likewise not available.

In light of the above issues, the *PLOS ONE* Editors retract this article.

SKS agreed with retraction. HS agreed with retraction and apologizes for the issues with the published article. PLC agreed with retraction, stands by the article’s findings, and apologized for the issues with the published article. S-HK did not agree with retraction and stands by the article’s findings. SX either could not be reached or did not respond directly.

Mock-infected panels of Figure 7A and 7B in [1] report material from [3], published 2011 by American Society for Microbiology, which are not offered under a CC-BY license and are therefore excluded from this article’s [1] license. At the time of retraction, the article [1] was republished to note this exclusion in the Figure 7 legend and the article’s copyright statement.
